# Phosphor-Necrosis

**Published:** 1872-06

**Authors:** J. E Garretson, De F. Willard


### ARTICLE V.

### *Phosphor-Necrosis.*

Service of J. E Garretson, M. D.

REPORTED BY DR. DE F. WILLARD.

There is, gentlemen, a disease of the maxillary bones which is found only in those persons engaged in the manufacture of lucifer matches; and, as instances are but rarely presented to this clinic, I will occupy the major portion of the hour in the consideration of the case before us.

The patient is a woman 45 years of age, who informs us that she has been engaged in the business for fifteen years, a portion of the time having been spent in Germany, the remainder in this country. Her symptoms are so typical that we may save time by deferring their enumeration until we reach them in the regular course of delineation.

The disease is a form of necrosis, caused by oxidized phosphorus introduced into the system by dissolution in the saliva, uniting perhaps with one of the alkaline salts which there so constantly abound. These fumes consist of phosphorus and phosphoric acid—the former being easily convertible into the latter by simple admixture with air—hypo-phosphorus acid, phosphoretted hydrogen, and possibly even a little of the pure vapor of phosphorus itself.

To these dangerous fumes the dippers and dryers in such establishments must necessarily be exposed, to the manifest risk of their health. But you will ask, "Is there no prophylactic measure which can be employed?" There is one very important one, namely, the constant watching and filling of every carious dental cavity; for it is stated that this disease never occurs except in persons of carious teeth, a statement readily explicable when we remember that such cavities would open a more direct route to the pulp, and thus to the periosteum. Attention, then, to such treatment of the teeth should constitute the first element in prevention. The second is scarcely less important. It consists in the use of a *respirator*, so attached to the head that no air can enter the nose or mouth without passing through a cloth diaphragm saturated with a solution of carbonate of lime or soda, or through a layer of sulphate of soda and slacked lime. A small amount of ammonia in the atmosphere would be a useful adjunct, as would also the daily administration of a teaspoonful of carbonate of magnesia internally.

Precisely why phosphorus attacks these bones in preference to others, I am unable to say, barring the dental explanation of ingress; neither can I give you any more satisfaction in regard to the method in which it produces its destructive action. The facts exist, and Salter, I believe, has suggested in explanation that the formation of a superphosphate incrustation may be incompatible with bone life.

The first symptoms are scarcely distinguishable from those of ordinary caries or necrosis. They are, first, toothache in the carious organ, then periodontitis, lifting the tooth in its socket to such an extent that occlusion of the jaws gives great pain, then swelling and puffiness of the gum, gradually extending to the cheek, which swelling is not open and frank as in ordinary inflammation, but is debased and unhealthy in all its appearances. The bone dies, pus is discharged, riddling the gums or burrowing through the cheek and down the neck; the teeth loosen and fall out, and the general constitution becomes seriously affected. In the lower jaw this destructive process usually progresses, regardless of interference, until the whole body is destroyed the rami alone remaining; but in the upper maxilla the line of demarkation is not apt to be so definite, and it is more difficult to prognose as to the extent of its ravages. It is, however, more under the control of the surgeon, yet exhibits a decided tendency to recur, unless the constitution is fortified.

The process of death and separation of any phosphor-sequestrum is exceedingly slow, occupying a period of from one to twelve months, or even longer, during which time the patient is not only subjected to the severe tax upon the constitution, but has also to endure an excessively offensive discharge, which not only poisons the very air he breathes, but also passes into his stomach, destroying the appetite and rendering life miserable.

Meanwhile, the periosteum, which seems to resist the enemy longest, has not been idle, but has made an attempt to wall off or enclose this dying portion with new bone, so that by the time the sequestrum is ready to be cast off it lies in a gutter of osteophytic incrustation, which does not, however, completely cover it in above, and thereby permits a more ready internal escape.

The patient before you is now passing through the process described. The earlier symptoms have all disappeared and she has now, as you see, a lower jaw which is greatly thickened and diseased, while the edentulous gum is a mass of spongy, sinus-riddled, angry-looking, sloughing tissue. I feel dead bone at every point; yet the sequestrum is not ready for removal. But you may ask, "How do I know that it is not ready?" In the first place, the disease has not been of long enough duration; and secondly, I find the bone immovable. But here let me warn you that a sequestrum may be so caught and held in the gutter of new material that it is frequently retained long after separation has really occurred. When this is the case, the bone, though apparently firm, yields before strong pressure, and is springy.

Such are some of the features of this justly dreaded disease. Once established, can we arrest it by treatment? Usually, we cannot. A certain portion of the bone—especially in the lower maxilla—is inevitably doomed; yet we can do much in the way of supporting the patient under the great drain to which he is subjected, hastening the process of death and separation, and—in the upper jaw—diminishing or circumscribing the advancing disease.

In all cases of toothache occurring in match-makers, then be upon your guard, seeing that carious cavities are immediately filled and the before-mentioned prophylactic measures employed. If seen in the acute inflammatory stage of periodontitis, the diseased and elevated tooth may be protected from the opposing organ by moulding over some opposite prominent *dent* a little cap of softened gutta-percha, which, when hardened by cold water, will receive the first stroke of the closing jaws and prevent further irritation. Active antiphlogistic measures must then be adopted, and—most important of all—the underlying periosteum and bone immediately relieved from tension by full free incisions made boldly down through the gums until the osseous structures are reached. At the same time it is wise to remember that the phosphorus poison may yet be susceptible of partial neutralization by means of carbonate of magnesia, which should be given to the amount of a teaspoonful twice a day, the same powder being also constantly applied locally to the diseased spot. I say susceptible of partial neutralization; for I do not believe that any known treatment will cause a complete arrest.

Constitutional measures should also be vigorously employed, since it is only at the outset that any benefit may be expected from attempts at resolution. Iron, quinine, cod-liver oil, and stimulants may be urgently pushed for a few weeks; but if no improvement is perceptible after a month's trial, it is better to decrease them and depend mainly upon good air, food, and exercise, since it is not advisable to load a patient's stomach with drugs for ten or twelve consecutive months. In the later stages, the time will again arrive for their employment being needed to support the strength, and perhaps save the life of the patient.

During the progress of the disease, free incisions should be made in the gums, in order to give vent to the pus—to save integumental offerings, which are difficult to cure—

and to allow frequent syringings, with dilute carbolic acid, Labarraque's solution, or, better than all, the compound tincture of capsicum, with an excess of myrrh and the addition of a little permanganate of potash. In those terrible cases where extensive sloughing occnrs, it may be necessary to employ a mop to draw away these discharges and prevent the pus from passing into the stomach.

I have told you that the periosteum resisted the onslaughts of the disease longer than the bone isself; it has therefore been my practice for a number of years to hasten the process of bone-sloughing by inserting into the wound little pledgets of cotton, which insinuating themselves into its depths, expand, and slowly work off the already separating periosteum, thus leaving it bare and unnourished. You may say that this would only tend to render the sequestrum even larger than if left to its normal course; but I assure you that such is not the case. A portion of the bone is already, as has been remarked, predestined to die, and by this treatment such death is markedly hastened, thus saving weeks of exhaustive drain, and much valuable time, to the patient.

Cleanliness is all important. In the later stages, the most generous diet—which must be largely liquid—the strongest tonics, the purest stimulants, may even fail to prevent the vital force from yielding, and the patient sinks under continuous hectic, tuberculous complications, or extensive sphacelus.

When hemorrhages occur and exhaust the patient, they should be controlled by the administration of tinct. erigeron. canaden., gtt. ij twice a day, or oftener if necessary.

The teeth should be removed as fast as they loosen, since if retained, they but irritate the cheek and gums.

Such should be your treatment—your highest duty being performed in sustaining the constitution and in waiting patiently for separation, be it one or fifteen months. *Early operative interference is only productive oj harm*, for, even if not followed by death, an inflammatory sequestrum usually forms, which is but an additional drain. I remember well a girl whom I treated in this city for months, warning her from the outset that she must have unbounded faith and patience, two mental properties, by the way, which are exceedingly difficult to maintain when one sees herself, month by month, becoming worse and worse, and with no encouragement for the immediate future. At about the seventh month, although progressing as well as possible, the patient became restive, and, finding that I persisted in my determination not to cut away the sequestrum, sought other advice. The jaw was resected through the rami, and in ten days she was in her grave. She died of pyæmia, or shall I say blood-poisoning? And why? Because that bone was in such a soft porous condition, between life and death that it sucked up the pus as readily as a sponge imbibes the water. I cite this case because it is but a mirror of my personal experience, and, although it is in opposition to the views of many Continental surgeons, yet I feel that my words will be found true,—as regards American cases, at least.

When, however, the proper time has come, and the sequestrum is loose—as you will determine by frequent trials—you can lift it away with but little risk; and a mass of new material will remain, which, though subsequently undergoing a certain amount of absorption, will always assist greatly in mastication, and preserve the contour of the face with but slight deformity. External incisions are seldom necessary, even for the removal of an entire jaw; the external openings requiring only to be enlarged to the adequate extent. It is easier to take away the lower jaw by sections so that it becomes necessary to split it at the symphysis with chisel, saw, or, preferably, cutting bone-forceps. Once divided, the pieces can be lifted without trouble, unless held by overlying osteophytic structures or indurated soft parts, which circumstance will be readily recognized by the springy impression imparted to the hand.

Even when the entire jaw is removed, the remaining new structure forms a sort of ligamentous attachment to the temporal bone, which allows of joint-movement.

When the dead bone is thus removed "at full term," as we may call it, there is but little danger of a return of the disease, provided due care be used. The occupation should be changed for an out-door one, good diet secured, and frequent syringings practiced with water medicated by iodine or creosote.

In my remarks I have been speaking with especial reference to the lower jaw. In the upper one, I will say that necrosed portions may be removed at a much earlier period, since there is no definite point at which the disease will cease, and there is less danger of pyæmic poisoning. The tendency to recurrence is, however, very great.

The patient was put upon the plan of treatment above indicated, being directed to return at frequent intervals, and also warned that no immediate improvement need be expected.—*Med. Times.*

---

